# Crystal structures of wild-type and mutated cyclophilin B that causes hyperelastosis cutis in the American quarter horse

**DOI:** 10.1186/1756-0500-5-626

**Published:** 2012-11-08

**Authors:** Sergei P Boudko, Yoshihiro Ishikawa, Thomas F Lerch, Jay Nix, Michael S Chapman, Hans Peter Bächinger

**Affiliations:** 1 Research Department, Shriners Hospital for Children, Portland, OR 97239, USA; 2 Department of Biochemistry and Molecular Biology, Oregon Health & Science University, Portland, OR 97239, USA; 3 Molecular Biology Consortium, Advanced Light Source Beamline 4.2.2, Lawrence Berkeley National Laboratory, 1 Cyclotron Road, Berkeley, CA 94720, USA

**Keywords:** Peptidyl prolyl *cis-trans*-isomerase (PPIase), Cyclophilin B (CypB), Endoplasmic reticulum, Chaperone, Protein complex, Calreticulin, P-domain, Lysyl hydroxylase, Collagen, HERDA

## Abstract

**Background:**

Hyperelastosis cutis is an inherited autosomal recessive connective tissue disorder. Affected horses are characterized by hyperextensible skin, scarring, and severe lesions along the back. The disorder is caused by a mutation in cyclophilin B.

**Results:**

The crystal structures of both wild-type and mutated (Gly6->Arg) horse cyclophilin B are presented. The mutation neither affects the overall fold of the enzyme nor impairs the catalytic site structure. Instead, it locally rearranges the flexible N-terminal end of the polypeptide chain and also makes it more rigid.

**Conclusions:**

Interactions of the mutated cyclophilin B with a set of endoplasmic reticulum-resident proteins must be affected.

## Background

Hyperelastosis cutis (HC) or Hereditary equine regional dermal asthenia (HERDA), a degenerative skin disease in Quarter horses has been reported since 1978, in which the skin is described as hyperelastic, fragile and thin, with slow-healing wounds characterized by atrophic scars [1]. Lesions may be single or multiple, and are most commonly seen on the dorsum, although they may also occur on the legs [1]. Foals affected with HC rarely show symptoms at birth, but develop seromas, hematomas, and ulcerations primarily along the dorsal aspect that progressively worsen in frequency and severity with age. Many cases are not identified until the horses have a saddle on their backs, and lesions are most commonly found along the dorsal aspect, coincident with where the saddle would rest. The majority of affected horses are euthanized. Published case reports describing HC have compared the disease to Ehlers–Danlos syndrome [2].

A missense mutation in cyclophilin B (CypB) has been identified and pointed to a causal candidate gene for HC [3]. The mutation alters a glycine residue that has been conserved across vertebrates. The mutation is homozygous in affected horses. Screening of control Quarter horses indicates a 3.5% carrier frequency [3]. CypB is a peptidyl prolyl *cis-trans*-isomerase (PPIase) found in the endoplasmic reticulum (ER) [4,5]. CypB plays a significant role in the triple helix folding of collagen [6]. It catalyzes the slow *cistrans* isomerization of multiple proline residues in the collagen chain. Severe *osteogenesis imperfecta* (brittle bone disease) was observed in patients with mutations in CypB [7] or in CypB-deficient mice [8]. The more prevalent autosomal dominant forms of *osteogenesis imperfecta* are caused by primary defects in type I collagen, whereas autosomal recessive forms are caused by deficiency of proteins like CypB which interact with type I procollagen during post-translational modification and/or folding [9]. CypB forms a complex with prolyl 3-hydroxylase and cartilage-associated protein [10]. The complex plays an important role in collagen biosynthesis [11]. Like other ER chaperones and foldases, CypB is involved in formation of transient multi-protein complexes that facilitate individual protein activities on nascent chains [12]. The binding partners of CypB are PDI (protein disulfide-isomerase), HSP47 (47kDa heat shock protein), BiP (binding immunoglobulin protein), GRp94 (94 kDa glucose-regulated protein), ERp72 (ER resident protein 72), ERp5 (ER protein 5), calreticulin, calnexin [13].

Here, we present the crystal structures of recombinantly produced wild-type and HC-mutant CypB from the horse. The only difference is within the N-terminal labile end that plays a role in complex formation with other ER-resident chaperones and foldases.

## Results

### Overall structure

**Figure 1:**
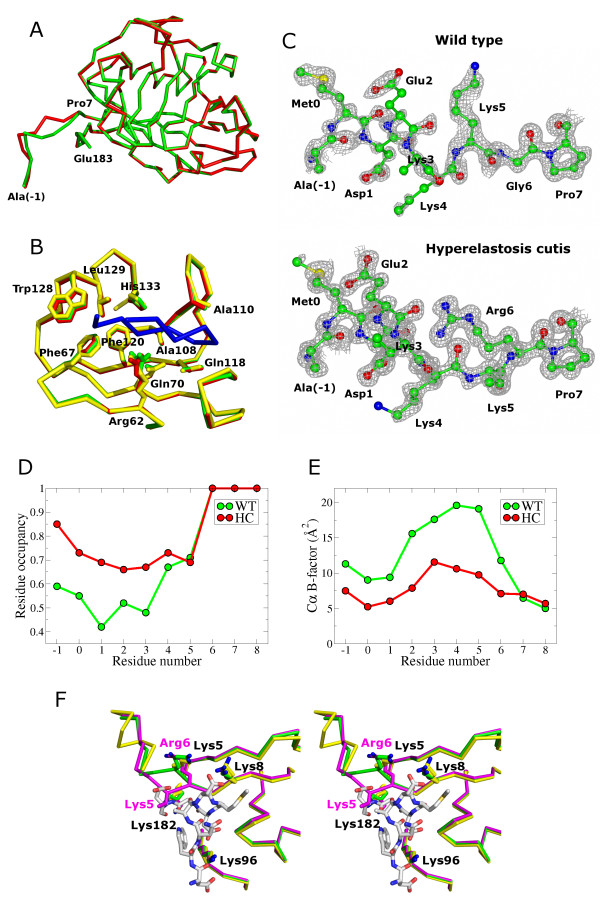
**Crystal structures of wild-type and HC CypB. A**. Cα ribbon presentation of horse wild-type (green) and HC (red) CypB. Chain displacement is observed within the N-terminal tail (residues from Ala(−1) to Pro7) **B**. Superimposed active site of horse wild-type (green), HC (red) and human (yellow; PDB: 1CYN [18]) CypB. Cyclosporin A is shown as ribbon (blue), which is a part of the complex with human CypB. Sidechains of residues that contribute to the binding of cyclosporin A are shown as sticks. **C**. Electron density maps for wild-type and HC CypB molecules calculated using 2Fo-Fc coefficients contoured at 1.0σ and shown in gray. **D**. Refined residue occupancy values of the N-terminal sequence for the wild-type (green) and HC (red) CypB models. **E**. Refined Cα B factors of the N-termini for the wild-type (green) and HC (red) CypB models. **F**. Stereo pair of superimposed structures of horse wild-type CypB (green), HC CypB (magenta) and the human CypB (yellow) complexed with the P-domain of calmegin [14] (white).

Wild-type and HC CypB were crystallized using the same crystallization conditions in space group C2 with one molecule per asymmetric unit. Therefore, all differences observed in the atomic models are attributable to the HC mutation (Gly6->Arg). Human and horse CypB share 96% sequence identity and the crystal structures are near identical (rmsd < 0.46 Å for 178 equivalent Cα positions, human CypB PDB: 3ICH [14]). The HC CypB molecule demonstrates the same overall structure with the exception for the very short N-terminal region that precedes the mutation site (Figure 1A) (rmsd = 1.31 Å for 8 equivalent Cα positions). The catalytic site remains undisturbed in the HC CypB molecule (Figure 1B).

### N-terminal region

Strong electron density is observed for all N-terminal residues of HC CypB (Figure 1C). While slightly weaker, electron density in this region was sufficient to unambiguously model the wild-type structure. In a previously reported high-resolution structure of human CypB the first three residues are disordered [14]. The presence of two recombinantly added amino acids (Ala-Met) preceding the natural sequence in our constructs of both HC and normal CypB accounts for a partial stabilization of the N-terminal segment via charge/polar contacts of Ala(−1) with His133 of a neighbor molecule (a zinc ion coordinates this interaction) and hydrophobic environment for Met0 formed by residues Met68, Ala108 and Phe120 of the adjacent molecule. The conformations of N-terminal sequences are only partially populated as revealed by the difference map, therefore variable occupancies were allowed for residues ranging from Ala(−1) to Lys(5) (Figure 1D). Moreover, weak difference densities from Fo-Fc maps in the vicinity of Ala(−1) to Lys5 suggest that the structure might adopt alternate conformations in this region, but only one conformation could be modeled. The N-terminal sequences appear to be the most flexible, with refined B-factors higher than average (Figure 1E). The Gly6->Arg mutation in HC CypB appears to endow greater stability in the N-terminal region, as residues Ala(−1) to Arg6 in the HC mutant structure have higher occupancy values for residues (~0.7 vs. ~0.5 for wild-type) and significantly lower refined B-factors (5–12 Å^2^ vs. 9–20 Å^2^ for wild-type). Thus, the Gly6->Arg mutation causes significant change in the structure (up to 4.2 Å shift for the backbone at residue Lys5), with the wild-type side chain density of Lys5 being partially occupied by HC Arg6, and the flexibility of the N-terminal region sharply reduced.

### P-domain interaction interface

The recently reported crystal structure of human CypB complexed with the proline-rich P-domain of calmegin reveals that the binding involves the N-terminal region of cyclophilin and the surfaces of CypB and P-domain are complementary [14]. That binding site is on the opposite side of CypB than the peptidyl prolyl *cistrans*-isomerase activity site. The N-terminal region forms part of the P-domain binding interface, contributing several hydrogen bonds. In HC CypB, the binding interface is altered: CypB overlaps with the P-domain, when superimposed, such that it could adversely affect binding (Figure 1F).

## Discussion

Our structures suggest a molecular rationale for the disease phenotype of the HC mutation in CypB of the American quarter horse. Evidence is accumulating that the N-terminal region of CypB interacts not only with calreticulin, but also with ER chaperones and foldases. Indeed, we have recently shown an altered interaction between HC CypB with the P-domain of calreticulin and loss of complex formation with lysyl hydroxylase 1 [15]. Interactions of CypB with ERp72 and GRp94 also require the N-terminal tail as confirmed by NMR experiments with the strongest chemical shifts observed for residues Lys3, Lys4, Lys5, Lys8 [13]. The same mode of CypB binding might be valid for BiP, PDI, ERp5 and calreticulin, where a polyacidic sequence similar to the one located in ERp72 was recognized [13]. The Gly6->Arg mutation in HC CypB has two consequences; first, it adds an additional positive charge to the polybasic stretch, which is involved in the binding of the polyacidic region in calreticulin and thus can enhance the affinity, second, it significantly distorts the geometry and flexibility of the N-terminal tail and thus can slow down, limit or even eliminate binding-competent conformations. As seen in Figure 1F a conformation of the HC CypB N-terminal tail is likely incompatible with P-domain interactions. Nevertheless, HC CypB binds the P-domain of calreticulin, but shows aberrant kinetics when compared with the wild-type; extra slow phases are observed for both association and dissociation [15]. The two consequences of the mutation in HC CypB have a synergistic effect on the overall P-domain binding. Binding of lysyl hydroxylase 1 to HC CypB is fully abolished [15], which establishes a more important role of the N-terminal tail and a stronger requirement for the N-terminal tail flexibility. Three lysine residues preceding the mutated glycine residue can be involved in N^ε^ acetylation, which was recently reported for CypB [16]. The mutation might affect acetylation and this could also cause changes in the repertoire and mode of interactions with other proteins.

## Conclusion

Hyperelastosis cutis is a connective tissue disorder caused by the Gly6->Arg mutation in cyclophilin B. The high resolution structures of the recombinant wild-type and mutated horse cyclophilin B provide evidence that the active enzymatic site is undisturbed and suggesting that the disease phenotype is caused by changes in the structure and flexibility of the N-terminal tail, altering interactions with other ER-resident chaperones and foldases.

## Methods

### Expression and purification of wild-type and HC cyclophilin B

The wild-type and HC CypB were recombinantly produced in bacteria and purified as described [15].

### Crystallization and datacollection

The purified recombinant wild-type and HC horse CypB were dialyzed against 10mM Tris/HCl, pH 7.2, and concentrated to about 6 mg/ml using an Amicon spin concentrator. The proteins were crystallized at room temperature using the hanging drop vapor diffusion method. For crystallization, 1 μl of the peptide solution was mixed with 1 μl of the reservoir solution containing 0.1M MES, 10mM ZnCl_2_, 10% glycerol and 28% polyethylene glycol monomethyl ether 550, pH 6.5. The crystals of hexagonal shape appeared within 2–5 days. The crystals were directly frozen in liquid nitrogen since the 10% glycerol / 28% PEG MME 550 sufficed as the cryo-protectant condition.

Data collection was performed using crystals flash-frozen to 100K on the “NOIR-1” detector system at the Molecular Biology Consortium Beamline 4.2.2 of the Advanced Light Source, Lawrence Berkeley National Laboratory.

### Crystal structure determination

The images collected were indexed, integrated and scaled using MOSFLM and SCALA from the CCP4 suite [17]. The AMORE program was used within the CCP4 suite [17] to find initial molecular replacement solutions. A crystal structure of human cyclophilin B (PDB: 1CYN) [18] was used as a search model. A single outstanding solution was generated by AMORE. Iterative cycles of model correction and refinement were performed using COOT [19] and PHENIX [20], respectively. Hydrogens were added and refined in riding positions. B factors of all atoms except hydrogens were refined anisotropically. Seven alternative side chain conformations were built for the models of both the wild-type and HC CypB. Two zinc ions were found to be involved in crystal contacts. Two short fragments of polyethylene glycol monomethyl ether 550 were fitted near each model. The quality of the models was assessed with program MolProbity (http://molprobity.biochem.duke.edu/). Data collection and refinement statistics are summarized in Table 1. Figures were generated with programs PyMOL (http://www.pymol.org) and CCP4MG [21].

**Table 1:** Summary of data collection and refinement statistics

	**Wild-type CypB (4FRU)**	**HC CypB (4FRV)**
Data collection		
Space group	C2	C2
*a, b, c* (Å)	64.9, 44.1, 60.6	64.8, 44.2, 60.1
*α, β, γ* (°)	90, 95.2, 90	90, 95.5, 90
Resolution^a^ (Å)	16.4-1.1 (1.16-1.10)	16.3-1.1 (1.16-1.10)
Measured reflections^a^	208,598 (10,718)	206,447 (10,544)
Unique reflections^a^	62,397 (5,316)	61,849 (5,304)
Redundancy^a^	3.3 (2.0)	3.3 (2.0)
Completeness^a^ (%)	90.4 (53.4)	90.2 (53.4)
Matthews coefficient (Å^3^ Da^-1^)	2.1	2.1
Solvent fraction (%)	41.8	41.1
I/σI^a^	9.8 (3.9)	12.4 (4.2)
Wilson plot B-factor (Å^2^)	4.2	4.4
R_merge_^a^(%)	7.8 (27.2)	5.2 (24.2)
Refinement		
R-factor (%)	11.6	11.9
R_free_ (%)	13.9	13.8
Protein/solvent atoms^b^	1472/300	1481/293
Rmsd. of bond lengths (Å)	0.007	0.007
Rmsd. of bond angles (°)	1.30	1.29
Average B value^c^ (Å^2^)	10.0 (7.8)	10.2 (7.2)
Ramachandran favored/allowed/outliers (%)	97.8 / 2.2 / 0	97.8 / 2.2 / 0
MolProbity score [22]	0.64 (100^th^ percentile)	0.90 (99^th^ percentile)

^a^Values in parentheses are for the highest resolution shell.

^b^Excluding hydrogen atoms.

^c^Values in parentheses are for macromolecule atoms.

### Availability of supporting data

The atomic coordinates and structure factors have been deposited with the protein Data Bank (PDB: 4FRU for wild-type and 4FRV for HC CypB).

## Abbreviations

HC: Hyperelastosis cutis; CypB: Cyclophilin B; HERDA: Hereditary equine regional dermal asthenia; ER: Endoplasmic reticulum; PDI: Protein disulfide-isomerase; HSP47: 47kDa heat shock protein; BiP: Binding immunoglobulin protein; GRp94: 94 kDa glucose-regulated protein; ERp72: ER resident protein 72; ERp5: ER protein 5.

## Competing interests

The authors declare that they have no competing interests.

## Authors’ contributions

SPB, YI, and HPB conceived the study. YI purified and crystallized the proteins. JN collected the diffraction data. SPB solved the crystal structures, analyzed the data, and wrote the manuscript. TL and MSC helped with the diffraction data collection, analyzed the data and edited the manuscript. All authors read and approved the final manuscript.
